# Performance of cone beam computed tomography and conventional intraoral radiographs in detecting interproximal alveolar bone lesions: a study in pig mandibles

**DOI:** 10.1186/s12903-017-0390-5

**Published:** 2017-06-21

**Authors:** Vanessa Camillo Almeida, Lucas Rodrigues Pinheiro, Fernanda Cristina Sales Salineiro, Fausto Medeiros Mendes, João Batista César Neto, Marcelo Gusmão Paraíso Cavalcanti, Cláudio Mendes Pannuti

**Affiliations:** 1 Department of Periodontology, School of Dentistry, University of São Paulo, Av. Prof. Lineu Prestes, 2227, São Paulo, SP 05508-000 Brazil; 2 Department of Radiology, School of Dentistry, University of São Paulo, Av. Prof. Lineu Prestes, 2227, São Paulo, SP 05508-000 Brazil; 3 Department of Pediatric Dentistry, School of Dentistry, University of São Paulo, Av. Prof. Lineu Prestes, 2227, São Paulo, SP 05508-000 Brazil

**Keywords:** Periodontal diseases, Diagnosis, Dental radiography, Cone-beam computed tomography

## Abstract

**Background:**

Cone beam computed tomography (CBCT) has been largely used in dentistry. Nevertheless, there is lack of evidence regarding CBCT accuracy in the diagnosis of early periodontal lesions as well as the correlation between accuracy and lesion size. The aim of this study was to evaluate accuracy of CBCT and conventional intraoral radiographs in detecting different-sized interproximal bone lesions created in pig mandibles. The hypothesis was that CBCT accuracy would be superior to radiographs in detecting incipient bone lesions.

**Methods:**

Twenty swine dry mandibles were used, totalizing 80 experimental sites. Four groups were created according to exposure time to perchloric acid 70–72%: controls (no exposure), 2-hour exposure, 4-hour exposure, and 6-hour exposure. Standardized CBCT and conventional intraoral radiographs were taken and analyzed by two trained radiologists. The presence of lesions in the dry mandible was considered the gold standard. Sensitivity, specificity, and accuracy in detecting different-sized bone lesions were calculated for CBCT and intraoral radiographs.

**Results:**

Accuracy of CBCT ranged from 0.762 to 0.825 and accuracy of periapical radiography ranged from 0.700 to 0.813, according to examiner and time of acid exposure. Inter-examiner agreement varied from slight to fair, whereas intra-examiner agreement varied from moderate to substantial.

**Conclusions:**

CBCT performance was not superior to that provided by conventional intraoral radiographs in the detection of interproximal bone loss.

## Background

Computed tomography (CT) is an imaging modality that uses X-ray equipment to make cross-sectional pictures of the human body in any of the three spatial planes [1]. Since conventional radiographs present some limitations due to image overlay, what may make diagnosis and treatment planning confuse, dentists have become more interested in tridimensional images [2, 3]. As a result of its high diagnostic quality and lower radiation dose, when compared to fan beam tomography, cone beam computed tomography (CBCT) has been largely used in dentistry [3, 4]. Several studies have analyzed CBCT applications in implantology [5, 6], oral surgery [6–8], endodontics [9] and orthodontics [10]. Studies conducted with database images [11], human cadavers [12–19], swine mandibles [12, 20, 21] and patients with chronic periodontitis [22–24] have analyzed CBCT performance in diagnosis and treatment planning in periodontics. These studies suggest that CBCT is more accurate than periapical radiographs in detecting bone craters, dehiscence, fenestration and furcation involvement. Nevertheless, as regards one- or two-wall infrabony defects and horizontal bone loss, CBCT superiority over periapical radiographs remains controversial [12–16, 19, 21, 23], because some studies did not compare CBCT performance to periapical radiographs [19, 23]. Even when considering the studies in which comparisons were made, there was no unanimity in favor to CBCT.

The majority of these studies were conducted with human mandibles [12–19] and most of them used round burrs to simulate periodontal bone defects. However, burrs produce well-defined lesions that do not correspond to the pattern observed clinically [20] and its artificial presentation increases the examiner’s ability to detect bone lesions with CBCT [25]. In order to create realistic bone defects, two studies used perchloric acid as an alternative technique to the use of burrs [20, 26]. The acid runs into bone tissue producing irregular demineralization, whose shape can vary from incipient to extended lesions, according to the time of acid exposure. Some authors believe that CBCT seems to be a promising technique in the detection of initial periodontal lesions [17]. Nevertheless, there is lack of evidence regarding CBCT accuracy in the diagnosis of early periodontal lesions as well as the correlation between accuracy and lesion size. Therefore, the aim of this study was to verify CBCT accuracy in detecting simulated interproximal bone defects with different sizes when compared to conventional radiographs, using the parallel technique. Our hypothesis was that there would be a difference in favor of CBCT in detecting incipient defects, and that the difference between CBCT and conventional intraoral radiographs would be reduced when analyzing larger bone defects.

## Methods

### Mandible preparation

This project was approved by the Ethics Committee for Use of Animals in Research of the Institute of Biomedical Sciences of the University of São Paulo (Protocol CEP-ICB number 072/2012). Sample size calculation was performed considering a ROC curve of 0.9 compared to an ROC curve of 0.75. Adopting an alpha error of 5% and beta error of 20%, 75 experimental areas would be necessary. To compensate possible losses, 80 experimental areas were used, in 20 swine mandibles.

**Fig. 1:**
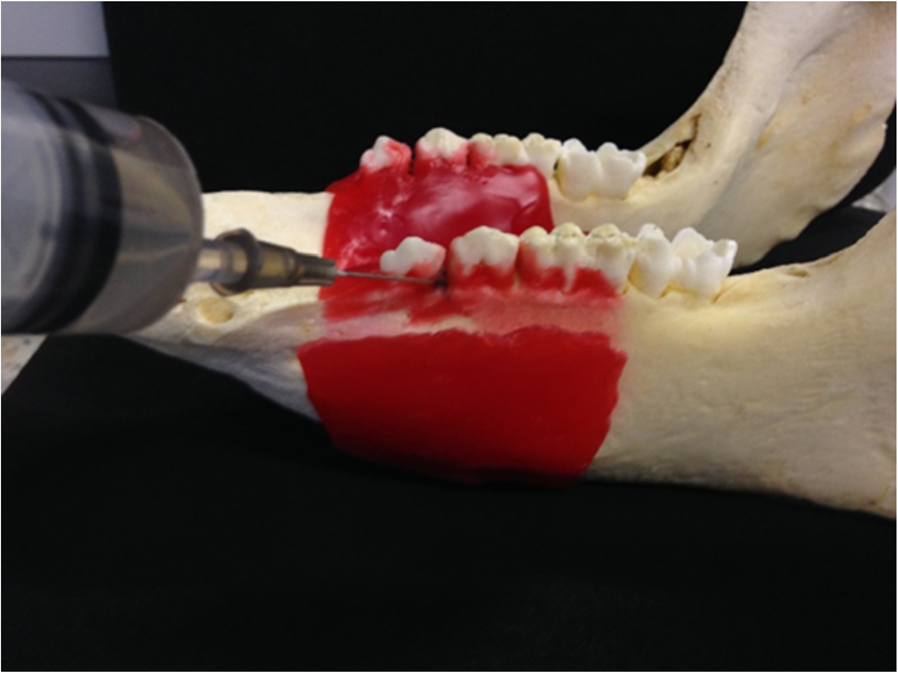
Swine mandible with *red* wax to simulate soft tissue and a cotton pellet being embedded with perchloric acid

Dry jaws were obtained from the files of the Department of Stomatology of the University of São Paulo. Red wax was used for soft tissue simulation [25]. Also, a small quantity of melted wax was applied to the proximal sides of teeth adjacent to the experimental areas in order to protect them from acid demineralization, since it could induce observers to guess in which areas lesions were created, even without noticing the bone loss. Figure 1 shows a swine mandible being prepared with the red wax and the perchloric acid.

Each pig jaw has 4 premolars (right and left PM1 and PM2) and 4 molars (right and left M1 and M2). Interproximal areas between PM1 and PM2 and between PM2 and M1 were considered experimental areas, totalizing 4 per mandible. In twenty sites, no lesions were created (controls). In sixty sites, lesions were created as follows: 20 with 2-hour acid exposure, producing lesions of 0.4 to 0.6 mm depth; 20 with 4-hour acid exposure, producing lesions of approximately 0.8 to 1.1 mm depth; and 20 with 6-hour acid exposure, producing lesions of approximately 1.8 to 2.2 mm depth. Acid exposure time was determined previously in a pilot study. Experimental time for each area was determined by a randomized sequence generator.

**Fig. 2:**
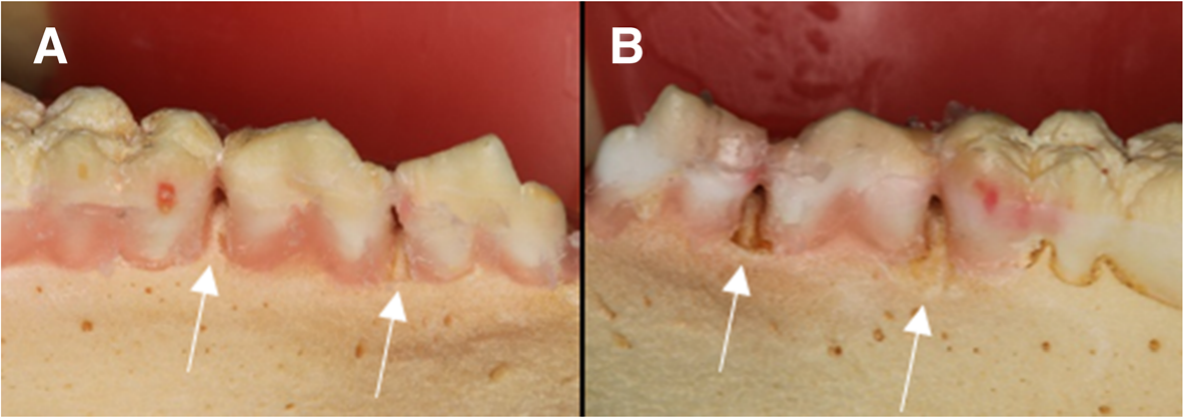
Swine mandibles with bone lesions. **a**. No lesion (control) between first molar and second premolar. Two-hour lesion between first and second premolar. **b**. Six-hour lesion between first and second premolar. Four-hour lesion between second premolar and first molar

The same researcher (VCA) who performed soft tissue simulation produced bone lesions. A cotton pellet was placed at the interproximal space, filling all the available space, an then soaked with perchloric acid 70–72% [20, 26] using a disposable syringe, until it was visible fulfilled. Cotton pellets were left in contact with bone for 1 h and replaced every hour until experimental times of 2, 4 or 6 h were achieved in order to produce standardized bone lesions with sizes varying from small to large. Figure 2a depicts a control site between first molar and second premolar and a two-hour lesion between first and second premolar. Figure 2b presents a six-hour lesion between first and second premolar and a four-hour lesion between second premolar and first molar. Once the experimental time was achieved, the site was washed and dried.

### Cone beam computed tomography

After preparing all mandibles, the same researcher (VCA) acquired CBCT images using a tomography equipment (ProMax 3D Max, Planmeca, Helsinki, Finland). Standardized position was achieved by positioning each mandible in a holder, which allowed occlusal plane to be parallel to the ground. Besides, positioning lasers allowed the placement of experimental mandibles in a position similar to that obtained with patient’s mandibles.

One operator was responsible for all CBCT acquisition and used standardized conditions and the following protocol: FOV for mandible premolars (5 x 5.5 cm), 96 kV, 11 mA, 12 s, voxel size of 0.1 mm and high resolution. This protocol generated an effective dose of less than 122 μSv [27].

### Conventional intraoral radiographs

The same researcher (VCA) submitted the same specimens to conventional intraoral radiographs, using the parallel technique with conventional films (Kodak Ultraspeed, Kodak, Rochester, NY, United States) and a standard intraoral X-ray machine (Gnatus, Ribeirão Preto, São Paulo, Brazil) operating at 60 kV, 7 mA for 0.32 s. This exposition time is shorter than clinically used to compensate the absence of soft tissue. All radiographs were taken by the paralleling technique and the object to the source distance was 30 cm. The positioner was fastened to mandible with a masking tape. The time-temperature method was used for image processing inside a dark room. The temperature of the solutions in tanks was 25 °C. All radiographs were processed simultaneously at the same holder and the protocol followed 2.5 min of development, 30 s of rinsing, 10 min of fixation, 20 min of washing and drying in a cabinet.

### Image analysis

Subsequently, two experienced and trained radiologists (LRP – examiner 1 – and FCSS – examiner 2) performed image analysis. Each image corresponded to a site and examiners did not know in which sites there was a lesion or not and, if there was a lesion, they were not aware of the time of acid exposure. They were asked to record presence or absence of lesion on the site observed in each image.

Examiners received codified images according to a randomized sequence. Consequently, they were also not aware of to which mandible the image belonged. The presence of lesions in the dry mandible was considered the gold standard. Each examiner made four independent evaluations: two for CBCT images and two for the conventional intraoral radiographs, with at least one-week interval, in order to measure reproducibility. The two examiners were asked to fill in a table 0 for absence of lesion and 1 for presence of lesion.

**Fig. 3:**
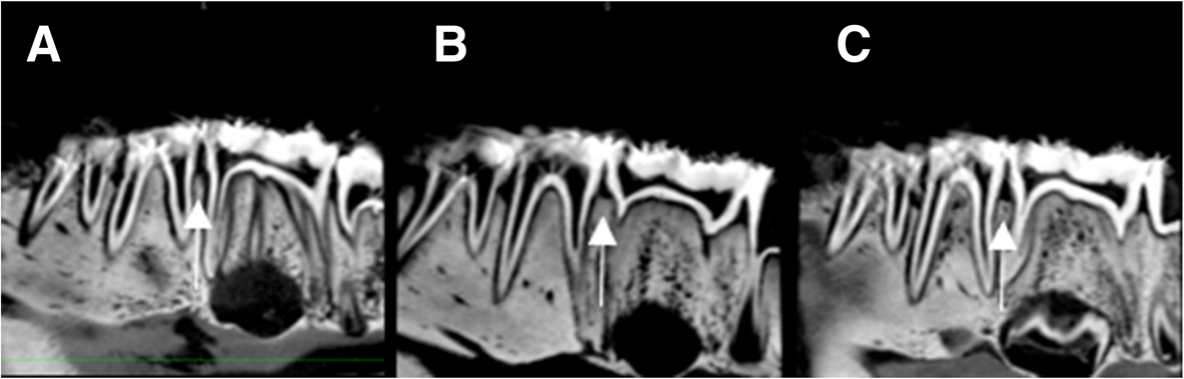
Sagittal sections of CBCT images. **a**. No lesion (control) between second premolar and first molar. **b**. Two-hour lesion between second premolar and first molar. **c**. Four-hour lesion between second premolar and first molar

**Fig. 4:**
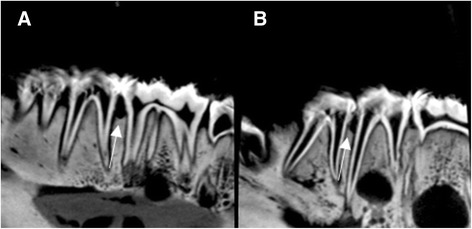
Sagittal sections of CBCT images. **a**. Crater formed after 6 h of acid exposure between second premolar and first molar. **b**. Vertical defect formed after 6 h of acid exposure between first and second premolar

**Fig. 5:**
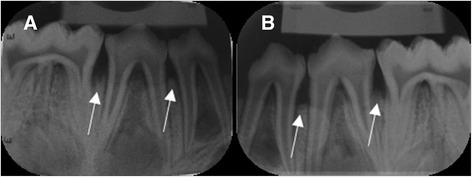
Periapical radiographs. **a**. Six-hour lesion between first molar and second premolar. No lesion (control) between first and second premolar. **b**. Four-hour lesion between first and second premolar. Two-hour lesion between second premolar and first molar

CBCT images were saved in DICOM standard format (version 3.0) and were imported to an open-source DICOM viewer (OsiriX 5.6, Pixmeo, Geneva, Switzerland), installed in an independent workstation (MacOS X v.10.6.8, Apple Inc., Cupertino, CA, United States). Analysis was performed independently and on separate occasions using an imaging post-processing protocol (OsiriX tool 3D-MPR, Pixmeo, Geneva, Switzerland). The images were analyzed simultaneously in axial, coronal, sagittal, parasagittal and circumferential views. Examiners were free to adjust brightness and contrast and to zoom in/out images, but not to apply filters. Figure 3a shows a sagittal section of control between PM2 and M1, 3B presents a two-hour lesion between PM2 and M1 and 3C shows a four-hour lesion between PM2 and M1. Figure 4a depicts a crater formed after 6-hour acid exposure between PM2 and M1 and 4B illustrates a vertical defect formed after 6 h of acid exposure between PM1 and PM2. Conventional intraoral radiographs were observed directly in a negatoscope inside a dark room. Examiners were free to use magnifying glasses. Figure 5a illustrates 6-hour lesion between M1 and PM2 and a control site between PM1 and PM2. Figure 5b shows four-hour lesion between PM1 and PM2 and two-hour lesion between PM2 and M1. There was no time limit for viewing radiographs or CBCT images.

### Statistical analysis

Inter- and intra-examiner reproducibility was measured by Kappa coefficient. Sensitivity, specificity and accuracy in detecting different-sized bone lesions were calculated for CBCT and conventional intraoral radiography, considering three different thresholds of detection: lesion produced by 2-hour acid exposure (site without lesion was considered as sound and lesions of 2, 4 and 6 h were considered affected sites); lesion produced by 4-hour acid exposure (sites without lesion and sites with 2-hour lesion were considered sound and lesions of 4 and 6 h were considered affected sites); and lesion produced by 6-hour acid exposure (sites without lesion and sites with 2- and 4-hour lesion were considered sound whereas lesions of 6 h were considered affected).

Percentage of correct answers to each type of site (without lesion and 2-, 4-, or 6-hour lesion) was also calculated to each imaging technique. McNemar test was used to compare CBCT and conventional intraoral radiographs, considering a significance level of 5%.

## Results

Table 1 shows first and second observations of each examiner using both methods divided by time of acid exposure. There was a tendency that the higher the time of acid exposure, the higher the number of right answers, both in CBCT and in intraoral radiographs. As regards control sites, examiners tended to provide more correct answers when using CBCT. There was a significant difference between the two techniques in the first examiner’s first analysis for 2-hour lesions (favoring radiograph) (*p* = 0.031) and 6-hour lesions (favoring CBCT) (*p* = 0.031). In all other examinations, there was no statistically significant difference between CBCT and conventional intraoral radiographs, representing similar performances for both techniques, independently of the examiner.

**Table 1:** Examiners’ analysis with both methods divided by time of acid exposure

	Control	2 hrs	4 hrs	6 hrs	Total
	N (%)	N (%)	N (%)	N (%)	N (%)
1st examiner – 1st observation					
CBCT					
No lesion	16 (80)	11 (55)	3 (15)	0 (0)	30 (37.5)
Lesion	4 (20)	9 (45)^a^	17 (85)	20 (100)^b^	50 (62.5)
Radiograph					
No lesion	13 (65)	5 (25)	6 (30)	6 (30)	30 (37.5)
Lesion	7 (35)	15 (75)^a^	14 (70)	14 (70)^b^	50 (62.5)
1st examiner – 2nd observation					
CBCT					
No lesion	16 (80)	9 (45)	5 (25)	2 (10)	32 (40)
Lesion	4 (20)	11 (55)	15 (75)	18 (90)	48 (60)
Radiograph					
No lesion	12 (60)	5 (25)	3 (15)	3 (15)	23 (28.8)
Lesion	8 (40)	15 (75)	17 (85)	17 (85)	57 (71.2)
2nd examiner – 1st observation					
CBCT					
No lesion	12 (60)	4 (20)	1 (5)	1 (5)	18 (22.5)
Lesion	8 (40)	16 (80)	19 (95)	19 (95)	62 (77.5)
Radiograph					
No lesion	9 (45)	4 (20)	2 (10)	0 (0)	15 (18.8)
Lesion	11 (55)	16 (80)	18 (90)	20 (100)	65 (81.2)
2nd examiner – 2nd observation					
CBCT					
No lesion	9 (45)	2 (10)	2 (10)	0 (0)	13 (16.2)
Lesion	11 (55)	18 (90)	18 (90)	20 (100)	67 (83.8)
Radiograph					
No lesion	10 (50)	2 (10)	2 (10)	1 (5)	15 (18.8)
Lesion	10 (50)	18 (90)	18 (90)	19 (95)	65 (81.2)

Equal letters indicate significant association between method and correct answers, calculated by McNemar test. (*p* = 0.031)

Table 2 presents inter- and intra-examiner reproducibility. According to Kappa coefficient [28], there was a fair inter-examiner agreement for CBCT analysis (0.21–0.40). However, inter-examiner agreement for intraoral radiographs analysis varied from slight (0.00–0.20) to fair (0.21–0.40). Examiner 1 showed substantial intra-examiner agreement (0.61–0.80) for CBCT and radiographs. Examiner 2 showed substantial intra-observer agreement for CBCT (0.61–0.80) and moderate agreement (0.41–0.60) for radiographs.

**Table 2:** Inter and intra-examiner reproducibility

Reproducibility	Kappa value (95% CI)	
	CBCT	Radiograph
Inter-examiner		
1^st^ assessment	0.362 (0.156 a 0.568)	0.200 (−0.005 a 0.405)
2^nd^ assessment	0.277 (0.087 a 0.468)	0.251 (0.021 a 0.481)
Intra-examiner		
Examiner 1	0.632 (0.457 to 0.806)	0.748 (0.597a 0.900)
Examiner 2	0.722 (0.530 a 0.913)	0.508 (0.266 a 0.750)

*95% CI* 95% confidence interval

Table 3 shows that conventional intraoral radiographs and CBCT had similar performances, not only for sensitivity and specificity but also for accuracy. In regard to sensitivity, CBCT varied from 0.767 to 0.933 whereas conventional intraoral radiographs oscillated from 0.717 to 0.917. In relation to specificity, CBCT varied from 0.450 to 0.800 whereas radiographs varied from 0.450 to 0.650. CBCT accuracy ranged from 0.762 to 0.825 and conventional intraoral radiographs accuracy extended from 0.700 to 0.813.

**Table 3:** Sensitivity, specificity and accuracy analysis for both methods (CBCT and conventional intraoral radiographs) divided by each examination of two radiologists

Methods	Sensitivity (95% CI)	Specificity (95% CI)	Accuracy (95% CI)
Examiner 1– 1^st^ analysis			
CBCT	0.767 (0.640 to 0.866)	0.800 (0.563 to 0.943)	0.775 (0.684 to 0.867)
Radiograph	0.717 (0.586 to 0.825)	0.650 (0.408 to 0.846)	0.700 (0.600 to 0.800)
Examiner 2– 1^st^ analysis			
CBCT	0.900 (0.795 to 0.962)	0.600 (0.361 to 0.809)	0.825 (0.742 to 0.908)
Radiograph	0.900 (0.795 to 0.962)	0.450 (0.231 to 0.685)	0.788 (0.698 to 0.877)
Examiner 1 – 2^nd^ analysis			
CBCT	0.783 (0.658 to 0.879)	0.700 (0.457 to 0.881)	0.762 (0.669 to 0.856)
Radiograph	0.817 (0.696 to 0.905)	0.600 (0.361 to 0.809)	0.763 (0.670 to 0.856)
Examiner 2 – 2^nd^ analysis			
CBCT	0.933 (0.838 to 0.982)	0.450 (0.231 to 0.685)	0.812 (0.727 to 0.898)
Radiograph	0.917 (0.816 to 0.972)	0.500 (0.272 to 0.728)	0.813 (0.727 to 0.898)

*95% CI* 95% confidence interval

## Discussion

The findings of the present study could not support the hypothesis of CBCT superiority over conventional intraoral radiographs in the detection of interproximal bone defects. As stated by the Swiss Society of Dentomaxillofacial Radiology at the Guidelines for the Use of Cone-Beam Computed Tomography/Digital Volume Tomography [29], there is still little information about the use of CBCT for periodontal diagnosis and treatment planning. As a tridimensional exam, CBCT offers potential advantages over intraoral radiographs. Hence, we wanted to evaluate if the use of CBCT as a tool for periodontal diagnosis would offer a great benefit for patients, keeping in mind its higher radiation dose.

In our study, CBCT accuracy ranged from 0.762 to 0.825, which is higher than Mol and Balasundaram [15] findings (0.74 accuracy in the detection of horizontal bone loss) and comparable to Noujeim et al. [17] results (0.77 accuracy in the detection of small bone defects created with burrs in the interproximal area of posterior teeth). In contrast to our results, Mol and Balasundaram [15] found CBCT superiority (0.74 accuracy) over periapical radiographs (0.48 accuracy) in the detection of horizontal bone loss in posterior teeth. Also, Vandenberghe et al. [16] observed that CBCT was more accurate than periapical radiographs when a 0.4 mm voxel was used. However, there was no statistically significant difference between CBCT and periapical radiographs when a 5.2 mm panoramic reconstruction was used. On the other hand, in another study from the same group [14], the authors did not find any significant difference between CBCT and periapical radiographs, when using 0.4 mm voxel to observe and measure natural horizontal bone loss in human mandibles. Also, Misch et al. [13] evaluated infrabony defects and affirmed that CBCT was as reliable as conventional intraoral radiographs.

Noujeim et al. [17] showed that accuracy of CBCT and periapical radiographs was higher in the detection of bigger defects (3 to 6 mm) than for small bone defects (1 to 3 mm). Also, Umetsubo et al. [20] affirmed that initial furcation lesions were more difficult to detect, regardless of the method used. In our study, we could also see a tendency of higher sensitivity for both techniques (CBCT and periapical radiographs) for bigger lesions, although the difference was not significant.

Besides accuracy, other factors must be considered for exam choice. Firstly, it is important to consider the risk-benefit to patient and all radiation protection principles must be followed. The principle of justification establishes that a patient should only be exposed to ionizing radiation after depleting all possibilities of clinical diagnosis or with the use of a method without radiation exposure. If an imaging exam is imperative, the principle of optimization must be followed, meaning that the magnitude of exposure should be kept As Low As Reasonably Achievable (ALARA).

Radiation dose of CBCT depends on application setting and technical specification selected during use (FOV, exposure time, kilovoltage and milliamperage) [30]. As a result, radiation dose of CBCT can vary from 29 to 477 μSv. On the other hand, a periapical radiograph produces around 5 μSv of radiation dose [31]. Furthermore, its cost is greater than intraoral exams and its interpretation requires training [17]. Our findings show that conventional intraoral radiographs can provide images as accurate as CBCT in the detection of interproximal bone defects. Thus, there is no evidence to support routine use of CBCT to detect interproximal defects as a preventive measure. Our results are in agreement with the guidelines of the Swiss Society of Dentomaxillofacial Radiology [29] and with a recent systematic review [32].

On the other hand, some authors suggest that CBCT may be an important tool in the diagnosis of furcation involvements [12, 14, 16, 19–22, 24], dehiscence [12, 18, 21] and fenestration [12, 21]. Also, CBCT may be an important tool in surgical planning [11]. We believe that the location of the bone defect, and not its size, is the key factor for finding CBCT benefits over conventional intraoral radiographs. CBCT may bring valuable information about buccal and lingual bone defects that may be missed by conventional intraoral radiographs, whereas both methods seem to be equally accurate as regard interproximal areas.

According to the model of imaging diagnostic efficacy proposed by Fryback and Thornubry [33], our investigation presents a level 2 diagnostic efficacy level. Level 2 is characterized by measures such as number of abnormalities found in a case series (presence of interproximal alveolar bone lesions), accuracy of diagnosis and measures of sensitivity and specificity. In this level of hierarchical model, both performance of the imaging method and the interpretation of the examiner are important. Interestingly, our results revealed the influence of the examiner in the interpretation of this imaging exam, since examiner 1 tended to underestimate diagnosis, pointing more negative results, whereas examiner 2 tended to overestimate diagnosis.

Further studies are needed to investigate potential new indications for CBCT. In addition, continuous studies are necessary, once this kind of technology is always being improved by manufacturers.

## Conclusions

CBCT performance was not superior to that provided by conventional intraoral radiographs in the detection of interproximal bone loss.

## Abbreviations

ALARA: As low as reasonably achievable
CBCT: Cone beam computed tomography
CT: Computed tomography
DICOM: Digital imaging and communications in Medicine
FOV: Field of view
M1: First molar
M2: Second molar
PM1: First premolar
PM2: Second premolar
